# *Agrobacterium*-mediated transient expression via root absorption in flowering Chinese cabbage

**DOI:** 10.1186/s40064-016-3518-1

**Published:** 2016-10-21

**Authors:** Lihua Zhong, Yuepeng Zhang, Houcheng Liu, Guangwen Sun, Riyuan Chen, Shiwei Song

**Affiliations:** College of Horticulture, South China Agricultural University, Guangzhou, 510642 China

**Keywords:** Agroinfiltration, *BcAMT1;3*, Flowering Chinese cabbage, Root absorption, Transient overexpression

## Abstract

**Background:**

Because most transient transformation techniques are inadequate for functional genomics research in roots, we aimed to develop a simple and efficient *Agrobacterium*-mediated transient transformation system that utilized root absorption for research in flowering Chinese cabbage.

**Results:**

Both semi-quantitative and fluorescent quantitative RT-PCR confirmed that the target gene *BcAMT1;3* was more highly expressed in plants that were infected with the transformed *Agrobacterium* strain (EHA105-p35S-*BcAMT1;3*) than in control plants that were infected with the control strain (EHA105-p35S). Furthermore, GUS staining analysis conformed the availability of this transient transformation system. In addition, we found that the highest transformation efficiency was achieved using an *Agrobacterium* cell density of OD_600_ = 0.3 for 3–6 h, without hyperosmotic pretreatment, and under these conditions, the peak transformation efficiency was observed at 2 and 4 d after infection.

**Conclusions:**

The transformation method developed by the present study is simple and convenient, since no special equipment is required, and since the method causes no damage, the plants can be used for subsequent experiments.

## Background

Functional genomics research often involves transgenic approaches that overexpress or silence genes, and among those approaches, both stable and transient transformation methods are valid for studying the functions and regulatory mechanisms of genes (Parinov and Sundresan 2000; Wroblewski et al. 2005). Compared to stable transformation, transient transformation is a more attractive alternative that allows transgenes to be assayed more rapidly and easily (Janssen and Gardner 1989; Kapila et al. 1997), so transient transformation has become the main strategy in functional genomics research and has been increasingly employed for research in various species (Ben-Amar et al. 2013; Bhaskar et al. 2009; Wu et al. 2014; Yang et al. 2008).

Various transient transformation methods have been developed. However, both polyethylene glycol-mediated transformation (Wilson et al. 1989; Yoo et al. 2007) and particle bombardment (Oard et al. 1990; Schenk et al. 1998) are complex and costly, since they require special devices, and virus-based methods (Fischer et al. 2013; Ratcliff et al. 2001) are limited to use in only a few plant species. As a result, *Agrobacterium*-mediated methods are used most widely and have facilitated research in various areas, including gene–gene interactions, gene silencing, gene regulation and expression (Gurlebeck et al. 2009; Ji et al. 2014; Johansen and Carrington 2001; Kim et al. 2007), and protein production (Barta et al. 1986; Benchabane et al. 2009). For *Agrobacterium*-mediated transient expression, leaf infiltration is the most common and efficient strategy used for *Agrobacterium*-mediated transient expression (Bhaskar et al. 2009; Voinnet et al. 2003). However, the method has limited applications for research involving roots, since the effects are mainly observed in leaves and because the method is incapable of investigating root-specific genes.

Flowering Chinese cabbage (*Brassica campestris* L. ssp. *chinensis* var. *Utilis* Tsen et Lee), whose edible organ is crisp flower stalk, is a subspecies variant of pak choi (*Brassica campestris* L. ssp. *chinensis* Makino var. *communis* Tsen et Lee), and one of the most widely cultivated vegetables in south China, and has the largest grown area and yield in local area (Song et al. 2012). Although both belong to a same species, there is great difference in morphology between flowering Chinese cabbage and pak choi, as the former is non-heading, and has flat green leaves and bolting flower stem. In addition, few transient transformation methods of flowering Chinese cabbage were reported, which seriously hindered its genetic research. Therefore, we aimed to develop a simple and efficient *Agrobacterium*-mediated transient transformation system that utilized root absorption for research in flowering Chinese cabbage and to optimize the key factors of transformation efficiency, including *Agrobacterium* cell density, transformation time, and hyperosmotic pretreatment conditions. As a result, a root-specific expression gene, ammonium transporter 1;3 of flowering Chinese cabbage (*BcAMT1;3*) was successfully overexpressed in root.

## Methods

### Plant materials

The experiments were conducted in the Horticultural Science greenhouse (25–30 °C, with natural sunshine) at South China Agricultural University. Seeds of flowering Chinese cabbage (*B. campestris* L. ssp. *chinensis* var. *utilis* Tsen et Lee) were sown in plug trays with perlite as a substrate, and after 3 weeks, the seedlings were transferred to hydroponic cultures in plastic pots. Each pot contained 12 plants and 24 L normal nutrient solution (4.0 mM NaNO_3_, 1.0 mM KH_2_PO_4_, 2.0 mM KCl, 1.0 mM MgSO_4_, 0.5 mM CaCl_2_, 0.1 mM Fe-EDTA, 50 μM H_3_BO_3_, 12 μM MnSO_4_, 1 μM ZnC1_2_, 1 μM CuSO_4_, and 0.2 μM Na_2_MoO_4_; pH 6.0, adjusted with 1 M NaOH or 10 % HCl), and 10 mg L^−1^ ampicillin was added to the nutrient solution to inhibit microbial activity. In addition, the growth solution was changed every 4 d.

### Plasmid construction

The 1515 bp CDS of the *BcAMT1;3* gene, including its stop codon, was amplified from the DNA of flowering Chinese cabbage roots using the following primers 5′-CACGGGGGACTCTAGAATGTCAGGACCTCTAACTTG-3′ (forward) and 5′-TCCTTTACCCATCCCGGGTTAAACGCGAGGAGGAGTAA-3′ (reverse). The sequence-verified amplicon was then cloned into the XbaI and SmaI sites of pBI121-35S vector using the In-Fusion HD Cloning Kit (TaKaRa Bio, Inc., Kusatsu, Japan), and the resulting constructs (pBI121-35S-*BcAMT1;3*) were verified by sequencing.

### Preparation of Agrobacterium suspension

The vectors pBI121-35S-*BcAMT1;3* and pBI121-35S were introduced into *Agrobacterium tumefaciens* strain EHA105 using the freeze–thaw method (Holsters et al. 1978). Single colonies of the *A. tumefaciens* strain EHA105-p35S-*BcAMT1;3* and EHA105-p35S were then grown in YEP medium (containing 30 mg kanamycin L^−1^ and 30 mg rifampicin L^−1^) at 28 °C with shaking. After overnight incubation, 1 mL of each culture was separately transferred to 50 mL of fresh YEP medium and incubated at 28 °C with shaking. When the culture density reached an OD_600_ of about 1.0, the cells were harvested by centrifugation at 5000 rpm for 10 min, and then adjusted to an OD_600_ of 0.6 in transformation solution (pH 6.0) that included the normal nutrient solution described above, 100 μM acetosyringone (AS), and 0.01 % (w/v) Tween20.

### Infection by root absorption

The root absorption infection procedure was modified from the method described by Ji et al. (2014). Briefly, the roots of plant seedlings were soaked in a hyperosmotic pretreatment solution of 20 % (w/v) sucrose (pH 6.0), and after 2 h, the roots were incubated in the transformation solution at 28 °C for 6 h with shaking at 120 rpm. Subsequently, the transformed seedling roots were washed with distilled water, transferred to normal nutrient solution containing 150 μM AS, and then sampled after 2 d for RT-PCR analysis.

### Optimization of the conditions of the transformation system

To optimize the transformation system, we also manipulated some of the transformation conditions, including the *Agrobacterium* cell density, length of transformation, sucrose concentration of hypertonic solution, length of pretreatment, and the use of both shaking and AS. The optimization experiments for each condition were performed independently, with each of the other conditions were described above. Then, under the optimized conditions, we sampled the transformed roots after 0, 2, 4, 6, and 8 d to determine the post-infection interval at which the target gene was most highly expressed.

### RT-PCR analysis

Total RNA was extracted using RNAiso Reagent (TaKaRa, Bio, Inc). To reduce the effect of plant-to-plant variability, a total of 24 plants were sampled from each treatment, and every eight plants were pooled together as one biological replicate. Subsequently, cDNA was synthesized from 1 μg aliquots of total RNA, using the PrimeScript RT Reagent Kit with gDNA Eraser (TaKaRa, Bio, Inc) in a reaction volume of 20 μL, and the synthesized cDNA was diluted 20 times with sterile water for use as the RT-PCR template. To determine the expression level of *BcAMT1;3*, semi-quantitative RT-PCR was performed using TaKaRa Ex Taq (TaKaRa, Japan), and fluorescent quantitative PCR was performed using a LightCycler 480 Real-Time PCR system (Roche, Basel, Switzerland) with SYBR Premix Ex Taq (TaKaRa, Bio, Inc). The primers 5′-TCGGAGAAGGATGAGATGG-3′ (forward) and 5′-CGAGGAGGAGTAACAGAACG-3′ (reverse) were used for amplification, and relative expression was calculated using the expression levels of two housekeeper genes, *ACTIN* and *GAPDH*, and the 2^−ΔΔCt^ method.

### Histochemical GUS Analysis

Reporter gene *GUS* was cloned into the pBI121-35S vector, and introduced into *Agrobacterium tumefaciens* strain EHA105, then the EHA105-p35S-*GUS* was transformed into flowering Chinese cabbage roots using the transformation system described above. GUS staining was carried out as previously described (Dong et al. 2001), and images of GUS-stained plants were obtained using a stereo light microscope (Leica, Germany).

## ^15^NH_4_^+^ uptake analysis

Ammonium (NH_4_^+^) influx assay was performed with flowering Chinese cabbage plants infected by EHA105-p35S-*BcAMT1;3* and EHA105-p35S, respectively. NH_4_^+^ influx measurements in *Arabidopsis* roots were conducted after rinsing the roots of hydroponically grown plants in 1 mM CaSO_4_ solution for 1 min, then to nutrient solution containing 0.2 mM ^15^N-labeled NH_4_^+^ (99 atom% ^15^N) for 5 min, and finally washed in 1 mM CaSO_4_ for 1 min. The influx solution was the same as used for plant growth except that 4.0 mM NaNO_3_ was replaced by 0.1 mM (^15^NH_4_)_2_SO_4_. Roots were separated from shoots and dried for 24 h at 65 °C. Samples were ground and approximately 1.0 mg of powder was used for ^15^N determination using isotope mass spectrometry (Thermo-Finnigan, Bremen, Germany).

### Statistical and graphical analyses

All data were statistically analyzed using one-way analysis of variance (ANOVA) and Duncan post hoc tests in SPSS 12.0 program (SPSS, Inc., Chicago, IL, USA). Graphs were produced using SigmaPlot 11.1.0 (Systat Software, Inc., Chicago, IL, USA), and all graphs and images were arranged using Adobe Photoshop 7.0.

## Results

**Fig. 1:**
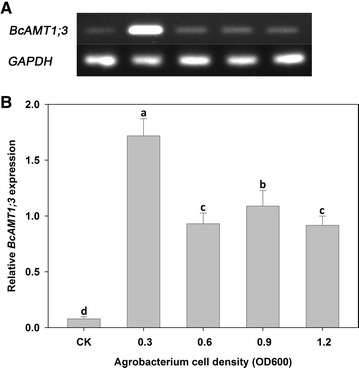
Effect of *Agrobacterium* cell density on the *Agrobacterium*-mediated transient transformation efficiency of flowering Chinese cabbage. The expression of *BcAMT1;3* was measured using semi-quantitative (**A**) and fluorescent quantitative (**B**) RT-PCR. The control (CK) column indicates the expression of *BcAMT1;3* in plants infected with the empty vector-containing EHA105-p35S *Agrobacterium*. Values are the mean ± SE (n = 3), and *different lower case letters* indicate significant differences among treatments (P < 0.05)

Both semi-quantitative RT-PCR and fluorescent quantitative real-time PCR confirmed that plants infected by EHA105-p35S-*BcAMT1;3* exhibited much higher expression of *BcAMT1;3* than plants infected by EHA105-p35S (CK), and the highest expression level was produced by treatment with an *Agrobacterium* cell density of OD_600_ = 0.3 (Fig. 1).

**Fig. 2:**
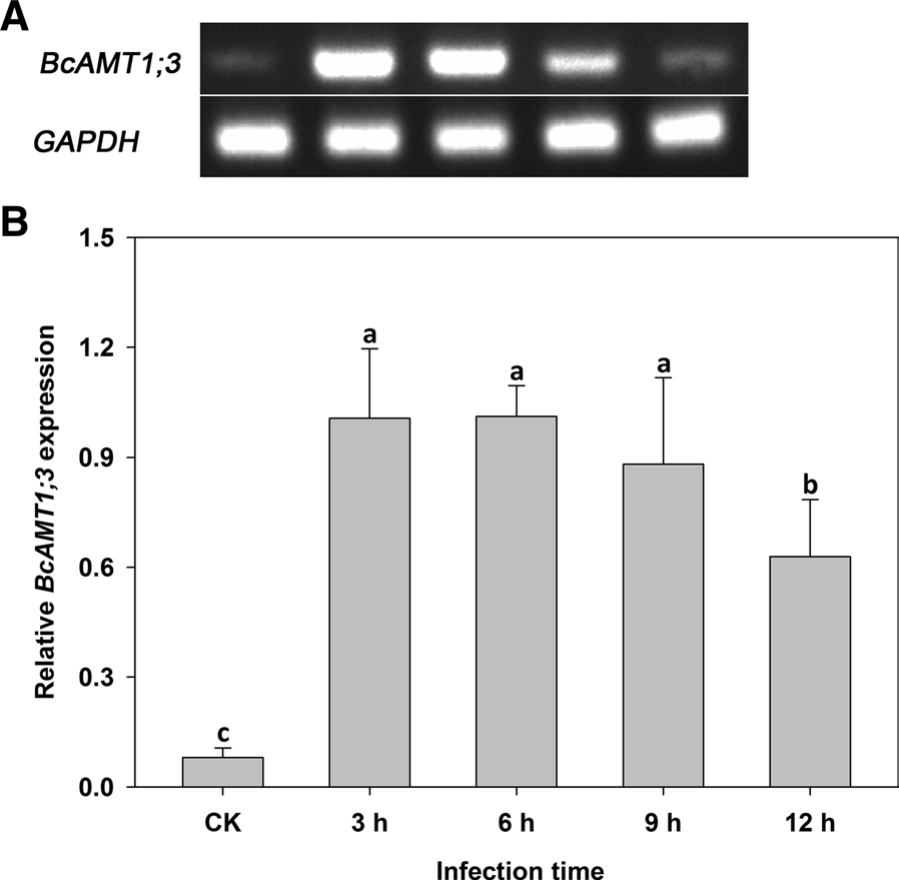
Effect of treatment duration on the *Agrobacterium*-mediated transient transformation efficiency of flowering Chinese cabbage. The expression of *BcAMT1;3* was measured using semi-quantitative (**A**) and fluorescent quantitative (**B**) RT-PCR. The control (CK) column indicates the expression of *BcAMT1;3* in plants infected with the empty vector-containing EHA105-p35S *Agrobacterium*. Values are the mean ± SE (n = 3), and *different lower case letters* indicate significant differences among treatments (P < 0.05)

By varying the duration of incubation in the transformation solution, we found that treatment for 3, 6, and 9 h yielded higher transformation efficiency, whereas treatment for 12 h was less efficient (Fig. 2). In this experiment, plants infected by *BcAMT1;3* exhibited much higher expression than the CK plants, too (Fig. 2).

**Fig. 3:**
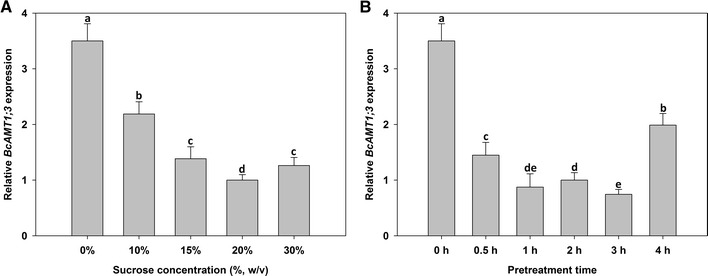
Effect of hyperosmotic pretreatment conditions on the *Agrobacterium*-mediated transient transformation efficiency of flowering Chinese cabbage. The response of *BcAMT1;3* expression to pretreatment with various sucrose concentrations for 2 h (**A**) or with 20 % sucrose for various durations (**B**) were measured using RT-PCR. Values are the mean ± SE (n = 3), and *different lower case letters* indicate significant differences among treatments (P < 0.05)

Higher *BcAMT1;3* expression was achieved when no sucrose pretreatment was used (Fig. 3), and the transformation efficiency decreased sharply with increasing sucrose concentrations (Fig. 3a) and pretreatment time (Fig. 3b). However, a slight increase was detected under pretreatment with 30 % sucrose for 2 h (Fig. 3a) and with 20 % sucrose for 4 h (Fig. 3b).

**Fig. 4:**
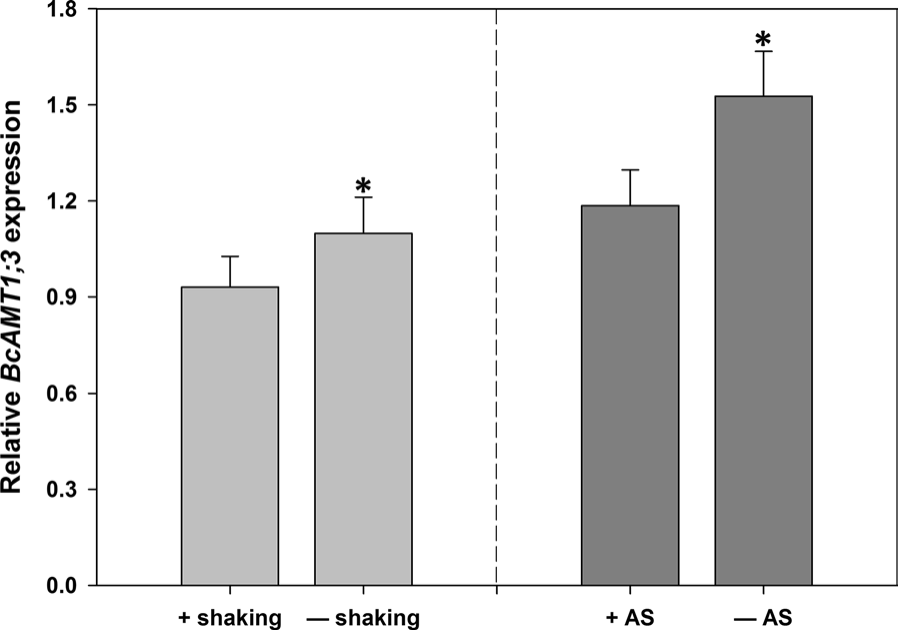
Effect of shaking and acetosyringone (AS) on the *Agrobacterium*-mediated transient transformation efficiency. “+shaking” and “–shaking” indicate the inclusion or omission of shaking during the infection procedure, respectively, and “+AS” and “–AS” indicate the presence or absence of acetosyringone (150 μM) in the nutrient solution after infection, respectively. Values are the mean ± SE (n = 3), *asterisk* indicates a significant difference between the two treatments (P < 0.05)

The incorporation of shaking during the infection procedure was not conducive to transformation, and the addition of AS to the post-infection nutrient solution reduced the transformation efficiency, as well (Fig. 4).

**Fig. 5:**
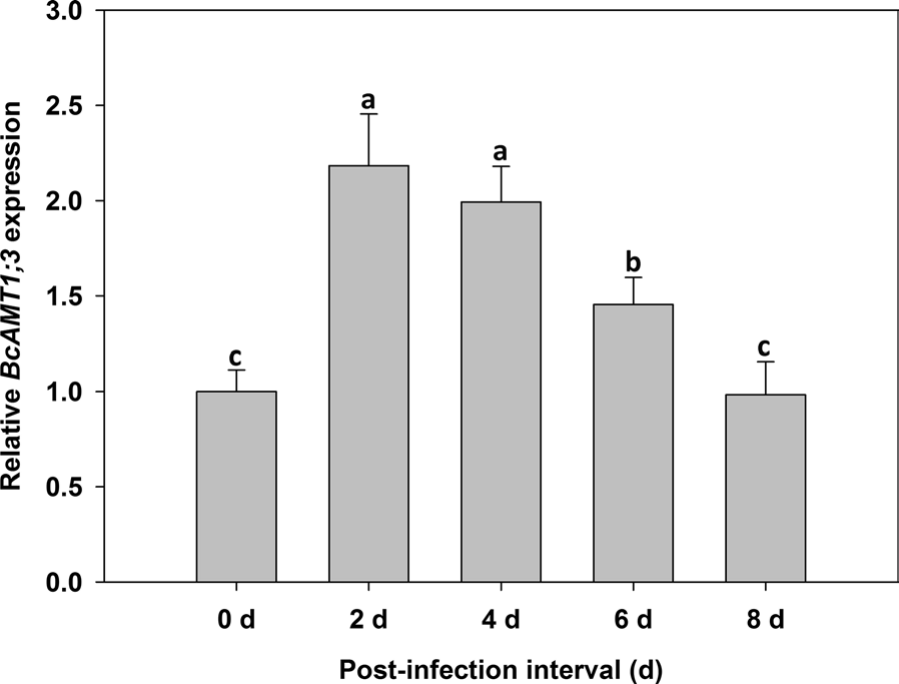
Effect of post-infection interval on the *Agrobacterium*-mediated transient transformation efficiency of flowering Chinese cabbage. The expression of *BcAMT1;3* was measured using fluorescent quantitative RT-PCR. Values are the mean ± SE (n = 3), and *different lowercase letters* indicate significant differences among treatments (P < 0.05)

Furthermore, we found that the transformation efficiency under ideal conditions (*Agrobacterium* cell density of OD_600_ = 0.3 for 3–6 h, without hyperosmotic pretreatment) peaked at 2 and 4 d after infection. Indeed, the level of *BcAMT1;3* expression was highest at 2 d after infection, and although the expression level gradually decreased, there was no significant difference between the level of expression at 2 and 4 d. Meanwhile. At 8 d after infection, the level of *BcAMT1;3* expression was indistinguishable from that at 0 d after infection (Fig. 5).

**Fig. 6:**
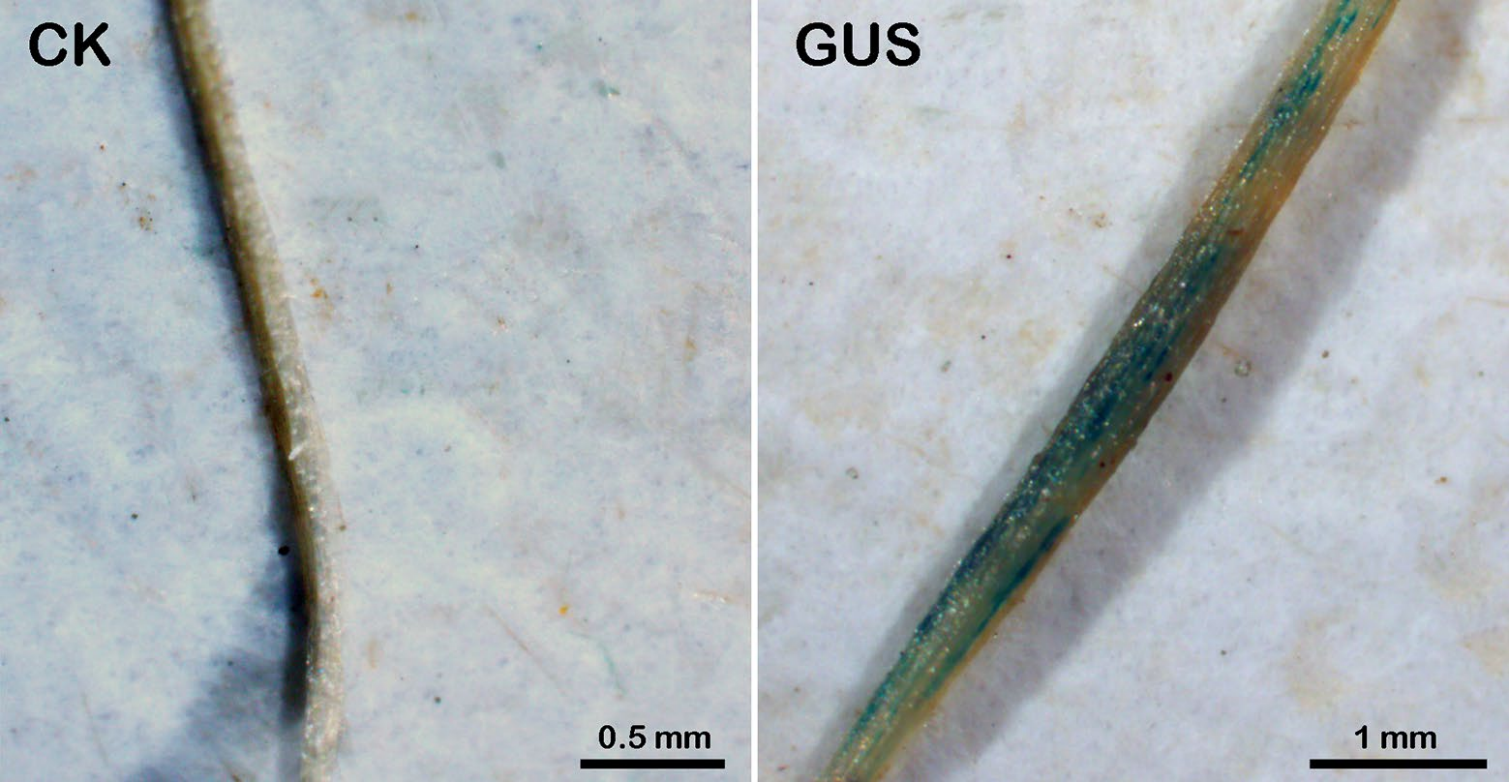
GUS staining analysis. GUS activity in roots was detected using the Leica stereoscope. “CK” and “GUS” in the figure mean GUS staining results of wild-type roots and EHA105-p35S-GUS infected roots, respectively

To further examine this transient expression system, the β-glucuronidase (GUS) reporter gene was transformed into wild-type flowering Chinese cabbage roots using the transformation system built above, and GUS activity in roots was detected using stereoscope. As shown in Fig. 6, GUS staining activity was detected in roots infected by EHA105-p35S-GUS, but not detected in the wild-type roots.

**Fig. 7:**
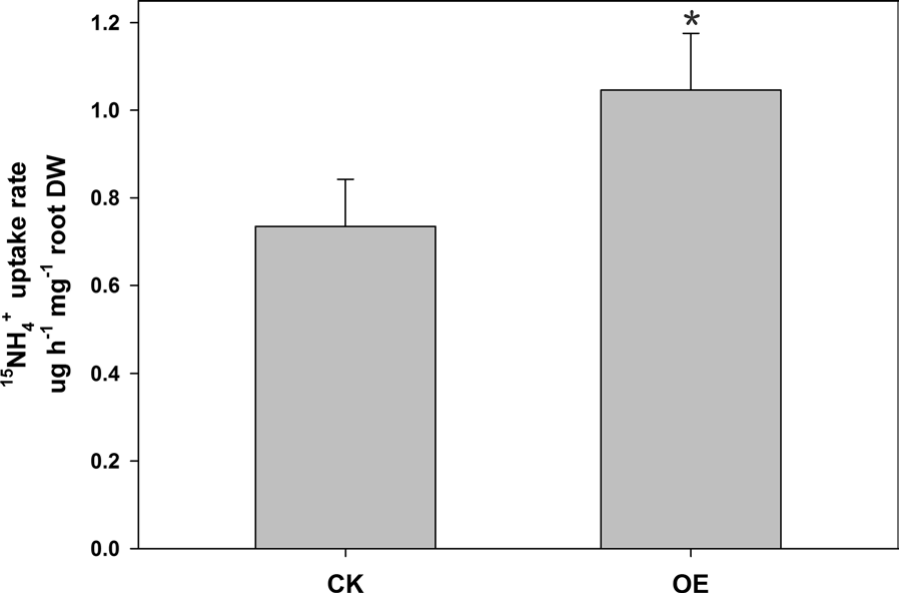
Effects of *BcAMT1;3* transient overexpression on ^15^NH_4_^+^ uptake. The CK and OE column indicates the ^15^NH_4_^+^ influx in plants infected with the empty vector-containing EHA105-p35S and EHA105-p35S-*BcAMT1;3 Agrobacterium*, respectively. Values are the mean ± SE (n = 5), and *asterisk* indicates significant difference the two treatments (P < 0.05)

AMT1;3 is an ammonium transporter, which has been proved functional in NH_4_^+^ transport across membranes (Gazzarrini et al. 1999; Loqué et al. 2006). Overexpressing *BcAMT1;3* in flowering Chinese cabbage roots significantly improved the influx of ammonium (Fig. 7), which also indicated that transient expression system in the present study is successful.

## Discussion

In the present study, we developed an *Agrobacterium*-mediated transient transformation system that utilized root absorption for research in flowering Chinese cabbage, and both semi-quantitative and fluorescent quantitative RT-PCR showed that plants transiently transformed with p35S-*BcAMT1;3* exhibited significantly higher expression of *BcAMT1;3* in roots (Figs. 1, 2). In addition, reporter gene *GUS* was transformed into flowering Chinese cabbage roots, and GUS staining activity detected in roots infected by EHA105-p35S-*GUS* further conformed the availability of this transient transformation system (Fig. 6). The method is both simple and convenient, because no special equipment was required, and moreover, since no damage is inflicted on the plants by vacuum infiltration or injection, the plants can be retained and used for subsequent experiments. We successfully examined the effects of *BcAMT1;3* overexpression of transient transformation plants on NH_4_^+^ absorption rate (Fig. 7). And in the case of Ji et al. (2014), for example, the method was used to detect the stress tolerance of transformed plants.

In the present study, we also optimized several key factors of transformation efficiency in flowering Chinese cabbage and found that infection with an *Agrobacterium* cell density of OD_600_ = 0.3 for 3–6 h, without hyperosmotic pretreatment, yielded the highest transformation efficiency. In the tobacco (*Nicotiana benthamiana*) root-absorption transient system used by Yang et al. (2008), the highest transformation efficiency was attained using an *Agrobacterium* cell density of OD_600_ = 1.23, which is more than four times greater than the optimum cell density for flowering Chinese cabbage, and in the *Tamarix hispida* root-absorption transient system, an *Agrobacterium* cell density of OD_600_ = 0.9 was used in all experiments (Ji et al. 2014). In the present study, higher *Agrobacterium* cell densities and longer infection times may have failed to yield better results, possibly because the roots of flowering Chinese cabbage are more tender than those of *N. benthamiana* and *T. hispida,* and young tissue composed of newly expanded cells from vigorously growing plants often exhibited higher levels of transient expression (Wroblewski et al. 2005). This could also explain why hyperosmotic pretreatment and shaking were not required either. In fact, we found that the leaves of transformed plants wilted after hyperosmotic pretreatment, which suggested that the hyperosmotic pretreatment might damage flowering Chinese cabbage roots.

## Conclusions

Root-absorption transient transformation systems have been developed in various species, including *N. benthamiana, A. thaliana*, *T. hispida, Betula platyphylla* (Ji et al. 2014; Yang et al. 2008), and, now, flowering Chinese cabbage; however, the conditions for transforming different species may differ. The transformation method developed by the present study is simple and convenient, since no special equipment is required, and since the method causes no damage, the plants can be used for subsequent experiments.
